# From genes to climate: a perspective on the importance of leaf shape

**DOI:** 10.1093/jxb/eraf421

**Published:** 2025-09-23

**Authors:** Gabriella Jessica, Mary E Byrne

**Affiliations:** School of Life and Environmental Sciences, The University of Sydney, NSW 2006, Australia; School of Life and Environmental Sciences, The University of Sydney, NSW 2006, Australia; University of Cambridge, UK

**Keywords:** CUC2, environmental response, gene regulation, KNOXI, leaf lobe, leaf serration, leaf shape, RCO, SPL9

## Abstract

Integrating knowledge on the regulatory genes controlling leaf shape with morphological responses to environmental variables has the potential to identify genetic loci that underlie plant adaptability to changing environments.

**Leaves occur in a vast array of sizes and shapes. This variation is determined by genetics, and is influenced by environmental conditions. In this viewpoint, we provide commentary on the opportunities available to integrate research on leaf shape with studies of plant responses to the environment. Such links can build our understanding of leaf shape evolution, and provide information on how genetic pathways involved in leaf development might expand or limit developmental plasticity in changing environments.**

Significant advances have been made to expand our knowledge of genetic factors that control leaf shape in model and non-model plant species. Recently, studies comparing closely related species have allowed predictions of evolutionary trajectories that have resulted in species-specific differences in leaf shape (Wilson-Sanchez *et al*., 2022). Much less is known about the physiological and ecological significance of leaf shape variation. However, several approaches are being used to study leaf shape in relation to plant physiology and geographic distribution across different climates. Here, we highlight several recent studies that advance our knowledge on the relationship between leaf shape and the global distribution of plants, as well as enhance our understanding of the fundamental genetic factors affecting leaf morphology. Integrating research on the genetics of leaf shape with studies of physiological performance in various environments has the potential to improve our understanding of ecosystem dynamics and predictions of plant adaptability to changing climates.

## Is leaf shape a reflection of climate?

**Fig. 1.**
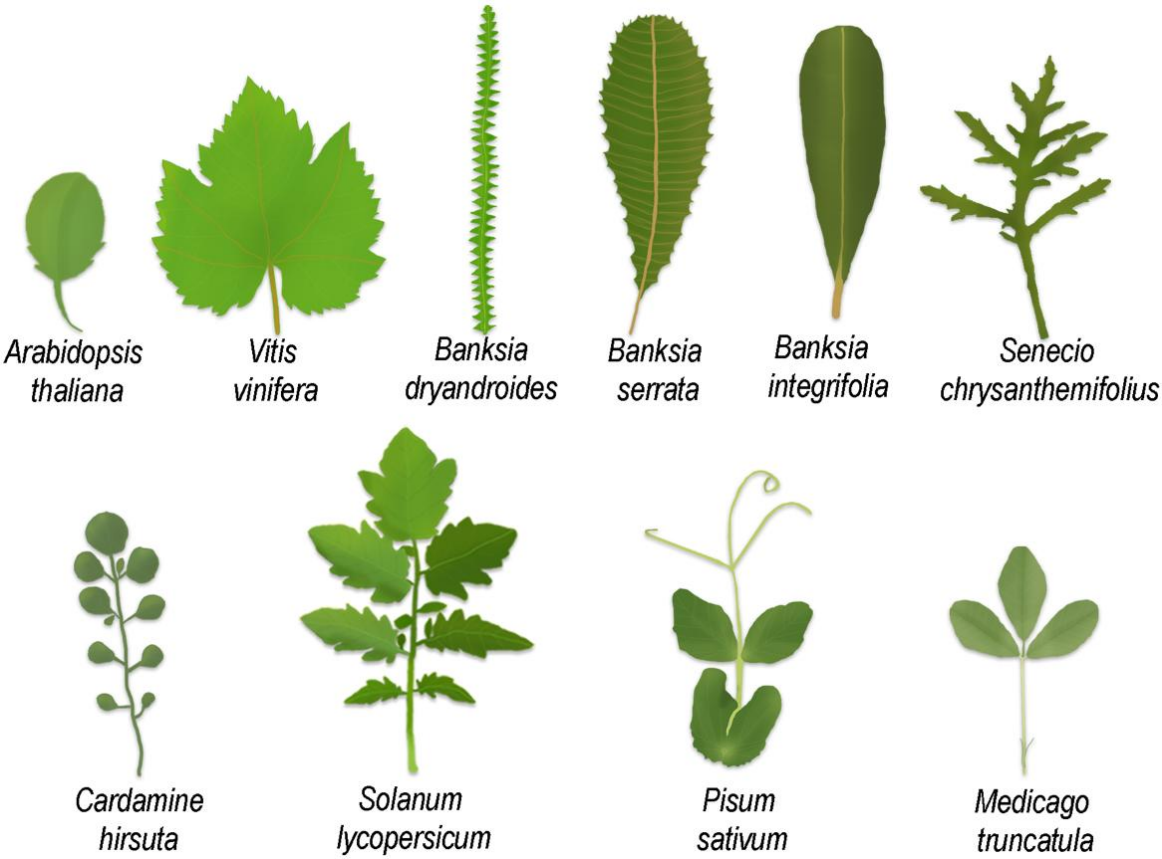
Leaf shape diversity across eudicot plants. Examples include simple serrated or lobed leaves of *Arabidopsis thaliana*, *Vitis vinifera*, *Banksia dryandroides*, *Banksia serrata*, *Banksia integrifolia*, *Senecio chrysanthemifolius* and compound leaves of *Cardamine hirsuta*, *Solanum lycopersicum*, *Pisum sativum*, *Medicago truncatula*. Leaf sizes are not to scale.

Leaf shape is a characteristic plant trait often used as a taxonomic discriminator of species. In an elementary description, leaves can be simple—with an undivided, continuous blade—or compound, with a blade divided into subunits called leaflets. Shape is further elaborated upon through growth along the leaf edge or margin, which may be smooth, or have patterns of outgrowths such as serrations or lobes (Fig. 1). Furthermore, leaf shape may vary over the lifetime of a plant, in a phenomenon called heteroblasty; and distinct populations of a species may display differences in leaf shape due to heterochrony, or differences in the rate of development (Poethig 2013).

Leaf shape can also vary in response to environmental conditions. A general relationship between leaf shape and distribution across different climates is well documented. Plants in cold climates typically leaf edges with increased number and size of serrations, and their leaves are highly dissected compared with the leaves of plants located in warmer, wetter climates (Peppe *et al*., 2011). A recent large-scale analysis of global tree distribution, which considered several leaf parameters, found that evergreen and deciduous trees with needle-shaped leaves are mainly found in colder boreal regions, whereas many broadleaved evergreen trees are found in warmer tropical regions, while broadleaved deciduous trees occur in a wide range of environments (Ma *et al*., 2023). Despite these generalizations, such connections are not universal, and different leaf shapes in related species do not always correlate with environmental conditions (Leigh *et al*., 2017).

Several non-mutually exclusive theories have been proposed to explain the physiological significance of leaf shape (Nicotra *et al*., 2011). Leaf shape affects heat, gas, and water exchange between the leaf and the atmosphere. Balancing these parameters is required to optimize photosynthesis and prevent tissue damage. For instance, lobed leaves have greater capacity for heat transfer and reach ambient temperature more readily compared with unlobed leaves. Lobed and dissected leaves also have a vascular pattern that has less hydraulic resistance stress than entire leaves, which might be advantageous in dry conditions (Nicotra *et al*., 2011). Leaf serrations are often associated with vascular tissue ends, or hydathodes, that may contribute to maintaining water and nutrient balance in the plant (Bellenot *et al*., 2022). The function of different leaf shapes in response to climate, which may help to explain global distribution patterns, is yet to be fully appreciated.

## Genetic mechanisms of leaf shape

**Fig. 2.**
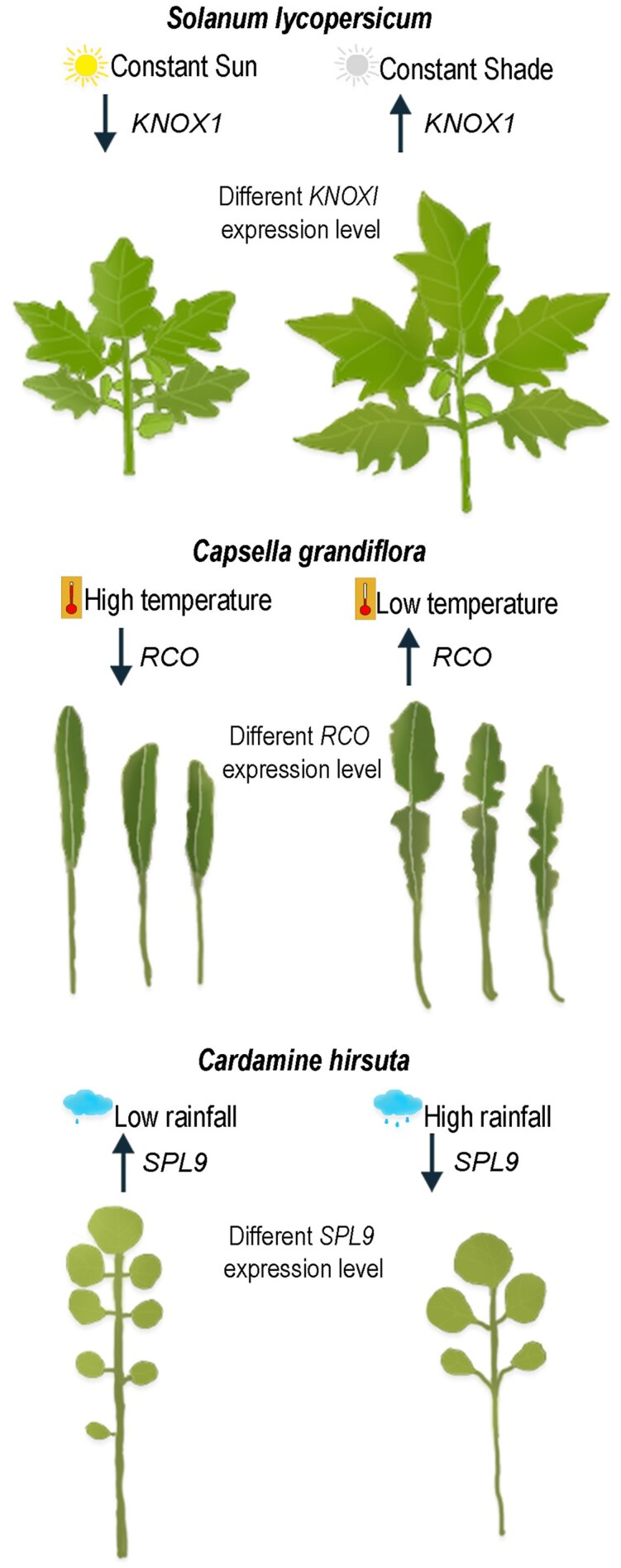
Environmental conditions influence leaf shape. Examples include light, temperature, and rainfall, which have been associated with alterations in leaf shape and changes in expression of key regulators of leaf shape in *Solanum lycopersicum*, *Capsella grandiflora*, and *Cardamine hirsuta*. Arrows indicate increased or decreased gene expression.

Despite considerable advances in our knowledge of the genetic mechanisms controlling leaf shape (Bhatia *et al*., 2023), this has yet to be fully translated into an understanding of the physiological impact of shape on plant growth and fitness. Here, we highlight several noteworthy genetic regulators of leaf shape, as there is strong evidence that these genes have played a significant role in the evolution of leaf shape, and are linked to responses to environmental conditions, suggesting that they may be of adaptive significance (Fig. 2).

*Class I KNOTTED1-LIKE HOMEOBOX* (*KNOX1*) genes have been studied extensively as key regulators of leaf shape. Across many angiosperms, *KNOXI* genes are not expressed in simple leaves, but are expressed in compound leaves (Bhatia *et al*., 2021; Byrne *et al*., 2024). When present, higher levels of *KNOXI* transcripts increase leaf shape complexity. In several species, *KNOXI* expression is linked with environmentally-induced changes in leaf complexity. For example, leaf complexity increases in response to shading in tomato and, to temperature changes in the aquatic *Brassicaceae* species *Rorippa aquatica,* with *KNOXI* genes up-regulated under these conditions (Fig. 2; Chitwood *et al*., 2015; Byrne *et al*., 2024). Despite this correlation, there has yet to be a systematic study across a broad range of plant taxa that addresses the extent to which leaf shape, climate, and geographic distribution correlate with the presence of KNOXI and relative levels of *KNOXI* gene expression.

A second notable gene controlling leaf shape is *CUP-SHAPED COTYLEDON2* (*CUC2*), encoding a transcription factor whose function is conserved across eudicot species with different leaf shapes. CUC2, along with auxin, forms a critical regulatory module required for serration outgrowth in *Arabidopsis thaliana* leaves, and for production of leaflets in *Cardamine hirsuta* (Bhatia *et al*., 2021; Byrne *et al*., 2024). *CUC2* is expressed at the leaf margin, where it promotes foci of auxin-mediated growth to form serrations, lobes, or leaflets (Blein *et al*., 2008; Bilsborough *et al*., 2011). Interestingly, in *Arabidopsis thaliana,* the prominence of leaf serrations depends on CUC2 protein level, which is determined by a regulatory network that includes an epigenetic component (Maugarny *et al*., 2024). CURLY LEAF (CFL) is a histone methyltransferase component of Polycomb Repressor Complex 2 (PCR2) that transcriptionally represses *CUC2* as well as *MIR164B*, a small RNA which acts as a post-transcriptional negative regulator of CUC2 levels. The epigenetic component of the system is proposed to provide a means for stable levels of CUC2 protein in various environmental settings, including light and temperature, and therefore robustness of leaf morphology (Maugarny *et al*., 2024). This raises the possibility that for some species, epigenetic mechanisms may provide phenotypic stability and a buffer against environmental change.

A third significant leaf-shape gene is *REDUCED COMPLEXITY* (*RCO*), which encodes a homeodomain transcription factor. *RCO* is crucial for leaflet formation in *Cardamine hirsuta*, with fewer leaflets forming in the absence of RCO (Vlad *et al*., 2014). *RCO* is one of a small cluster of 1–3 genes that, within the *Brassicaceae*, have evolved through gene loss, duplication, and neo-functionalization of duplicated genes, and through changes in expression and protein stability (Vlad *et al*., 2014; Vuolo *et al*., 2016). Higher levels of RCO also correlate with increased photosynthetic activity and plant productivity, suggesting that there may be adaptive advantages to the leaf shape conferred by RCO (Vuolo *et al*., 2016). RCO may provide genetic capacity for plants to adapt to different environments. Temperature-dependent variation in leaf margin lobing of several *Capsella* species can be traced to differences in the level of expression of *RCO* or *RCO*-related genes (Sicard *et al*., 2014; Streubel *et al*., 2018). Fitting with this data, an analysis of herbarium specimens of *Capsella bursa-pastoris* collected from across the United States indicated that leaf lobing was sensitive to seasonal temperature (Hightower *et al*., 2024). Variable RCO levels may explain some of the phenotypic plasticity and responses to temperature within *Capsella*. For example, *Capsella grandiflora* leaves are more lobed and have a higher level of *RCO-A* expression at lower temperature compared with that at higher temperature (Fig. 2; Sicard *et al*., 2014).

Lastly, we mention the *SQUAMOSA PROMOTER BINDING PROTEIN-LIKE* (*SPL*) transcription factor gene family. Several members of this gene family determine developmental transitions from juvenile to adult leaves, a function shared by multiple plant species. Such transitions can be associated with changes in leaf shape (Poethig and Fouracre, 2024). For example, in *Cardamine hirsuta*, the number of leaflets on successive leaves increases, and this change is driven by an increase in the expression of *SPL9* in successively older leaves (Li *et al*., 2024). A detailed study examining developmental variation and genetic structure in relation to geographic distribution of *C. hirsuta* natural populations found that heterochronic differences between populations could be mapped to genomic regions carrying SPL9. Furthermore, sequence polymorphism signatures provided evidence that the genomic region around SPL9 has been under positive selection. Populations of low or high leaflet number exhibit a clinal distribution across an island archipelago, which may be correlated with environmental differences such as rainfall (Fig. 2; Baumgarten *et al*., 2023).

## Does leaf shape reflect adaptation?

Leaf morphology is shaped by the interplay of genetic, evolutionary, and environmental factors. A significant question remains as to whether the characteristic leaf shape of a species, either alone or in combination with other parameters such as size, thickness, and vascularization, can be correlated with local climate and environment, and whether this has adaptive significance. Analysis of data from historic collections, common-garden experiments, and modelling ecological response dynamics, provide valuable approaches to assess the potential for plant population adaptability (Baumgarten *et al*., 2023; Ma *et al*, 2023; Walter *et al*., 2023; Hightower *et al*., 2024; Love and Ferris, 2024; Stroud and Ratcliff, 2025). Short-term transplantation and yearly seasonal studies have indicated a degree of phenotypic plasticity, including leaf shape features, that may be adaptive for growth under variable climates (Chitwood *et al*., 2016; Walter *et al*., 2023; Love and Ferris, 2024). However, the capacity for adaptation to rapid climate changes is likely to be species- and population-specific.

Our knowledge of the genetic basis of leaf shape variation, along with a capacity for targeted mutagenesis, provides a means to examine the impact of environmental conditions on plants with specific leaf-shape alleles in comparable genetic backgrounds. Such studies could be performed under common-garden or field-based conditions to assess performance. Combining field-based studies with genotype-phenotype research, along with the application of machine learning, has exciting potential to integrate plant development with ecology. This will enable us to better appreciate the evolution of phenotypic leaf shape variation, and provide greater scope for predicting plant species and population responses to climate change (Ferris, 2019; Stroud and Ratcliff, 2025). As we face rapid global environmental shifts, a holistic view linking leaf morphology, genetics, and the environment, may prove vital for maintaining plant diversity, as well as ensuring plant stability in different ecosystems (see Box 1). With a comprehensive understanding of leaf shape genetic determinants and physiological responses to changing ambient conditions, we might be able to optimize methods to limit plant diversity decline in natural ecosystems, and maintain more resilient plants in agriculture.

Box 1. **Questions for future research.**

- Does leaf shape correlate with macro or micro environmental conditions? Large-scale studies including multiple taxa, along with key environmental metrics could provide guiding rules placing leaf shape in ecological contexts.
- To what extent is leaf shape adaptive? Studies of geographically dispersed populations and transplantation experiments can provide information that correlates leaf shape with the environment.
- Can genetic-ecological studies provide predictive power to help maintain species diversity? Integrating biological genotype-phenotype data with population-based genetic and ecological studies could provide information to aid decisions in plant breeding and plant species conservation.
